# Season of birth and atopic dermatitis in early infancy: results from the Japan Environment and Children’s Study

**DOI:** 10.1186/s12887-023-03878-6

**Published:** 2023-02-15

**Authors:** Akiko Tsuchida, Toshiko Itazawa, Kenta Matsumura, Hiroshi Yokomichi, Zentaro Yamagata, Yuichi Adachi, Hidekuni Inadera, on behalf of the Japan Environment and Children’s Study Group, Michihiro Kamijima, Shin Yamazaki, Yukihiro Ohya, Reiko Kishi, Nobuo Yaegashi, Koichi Hashimoto, Chisato Mori, Shuichi Ito, Takeo Nakayama, Tomotaka Sobue, Masayuki Shima, Hiroshige Nakamura, Narufumi Suganuma, Koichi Kusuhara, Takahiko Katoh

**Affiliations:** 1 Department of Public Health, Faculty of Medicine, University of Toyama, 2630 Sugitani Toyama 930-0194, Toyama, Japan; 2 Toyama Regional Center for Japan Environment and Children’s Study, University of Toyama, Toyama, Japan; 3 Department of Pediatrics, Saitama Medical University, Saitama, Japan; 4 Department of Health Sciences, University of Yamanashi, Yamanashi, Japan; 5 Koshin Regional Center for Japan Environment and Children’s Study, University of Yamanashi, Yamanashi, Japan; 6 Department of Pediatrics, Faculty of Medicine, University of Toyama, Toyama, Japan

**Keywords:** Atopic dermatitis, Birth month, Birth season, Eczema

## Abstract

**Background:**

Atopic dermatitis (AD) is reported to be more prevalent in children who were born in autumn than in spring. Here, we investigated how early the association between season of birth and eczema or AD can be observed in the postnatal period. We also examined whether specific prevalence outcomes for infant eczema and AD differed according to sex and maternal history of allergic disease in a large Japanese cohort.

**Methods:**

Using data of 81,615 infants from the Japan Environment and Children’s Study, we examined the associations of birth month or season with four different outcomes—eczema at 1 month, 6 months, and 1 year of age and physician-diagnosed AD up to 1 year of age—using multiple logistic regression analysis. We also analyzed the effect of maternal history of allergic disease on these outcomes stratified by infant sex.

**Results:**

The risk of eczema at 1 month was highest in infants born in July. In contrast, infants born in autumn had higher risks of eczema at 6 months (adjusted odds ratio [aOR], 2.19; 95% confidence interval [CI], 2.10–2.30) and at 1 year (aOR, 1.08; 95% CI, 1.02–1.14) and of physician-diagnosed AD up to 1 year of age (aOR, 1.33; 95% CI, 1.20–1.47) compared with infants born in spring. Eczema and AD were more prevalent in infants with a maternal history of allergic disease, particularly boys.

**Conclusions:**

Our findings suggest that the prevalence of AD is associated with the season of observation. Eczema is prevalent in infants born in autumn, and this phenomenon was observed in infants as young as 6 months old. The risk associated with being born in autumn was particularly clear in boys with a maternal history of allergic disease.

**Trial registration:**

UMIN000030786

**Supplementary Information:**

The online version contains supplementary material available at 10.1186/s12887-023-03878-6.

## Background

Atopic dermatitis (AD) is a common allergic inflammatory skin disorder in children. Its etiology is not fully understood, but it is presumed to be multifactorial with interactions among genetic and environmental factors [1]. In terms of the genetic factors involved, the risk of developing AD is known to be higher if one parent has an allergic history than if neither parent such allergic history [2, 3], and mothers with AD may more readily transmit AD to their offspring compared with fathers [4, 5]. The sex of the child is also recognized to affect the development of allergic diseases. For example, boys are reported to be more susceptible to childhood asthma [6–8]. Nevertheless, the effects of sex on AD development, with and without maternal history of allergic disease, remain unclear.

In terms of the environmental factors involved, the severity of AD is known to change in relation to the seasons, and many people with AD in Japan experience exacerbations in winter [9]. A link has also been found between the prevalence of early childhood eczema and climate factors [10], with associations found with outdoor temperature and humidity [11]. Low indoor or outdoor humidity exacerbated subclinical eczema, thereby increasing the disease prevalence, through damage to the barrier function of the skin [10, 11]. Moreover, because rapid adaptations are required in the first few months of life after transitioning from the in utero environment to the very different *ex utero* environment, interest is growing in how prenatal as well as postnatal environmental exposures might contribute to AD risk in early childhood. Season of birth or month of birth can be used as a surrogate factor for potential environmental exposures during the prenatal and early postnatal periods. Factors that exhibit seasonal variation include temperature, humidity, dryness, exposure to sunlight, and outdoor physical activity [12, 13]. Studies of the relationship between season of birth and allergy have suggested that early infancy is of particular importance for the later development of allergic diseases [14, 15]. Many studies have reported that AD is more prevalent in children born in autumn than in spring in the northern hemisphere [14–22].

The International Study of Asthma and Allergies in Childhood (ISAAC) has developed a questionnaire for large-scale screening of AD and other allergic diseases in the general population [23, 24], but no widely used questionnaires have been developed to determine prevalence in infancy. Therefore, little information is available on the prevalence and development of skin lesions in the early postnatal period. Most previous studies assessed AD prevalence among children 6 years and older, and there are scant data on the prevalence of eczema and development of AD in younger children, especially those under 1 year of age. Up to 1 year of age, the diagnosis of AD is not common because the typical skin lesions are not obvious at that age. Moreover, physicians may not see children with eczema because over-the-counter medications are readily available to treat the symptoms. Yet, early eczema is regarded as the beginning of the atopic march [5, 25]. Indeed, atopic eczema is one of the earliest clinical manifestations of allergic disease [26] and occurs most commonly in the first months of life [27, 28]. It is important, therefore, to clarify how the season or month of birth affects the trajectory of the skin lesions in infants up to 1 year of age.

In this study, using data from a large Japanese birth cohort, we sought to clarify the effects of season of birth and maternal history of allergic disease on the development of eczema in infants up to 1 year of age. We wanted to determine how early in the postnatal period the association between the season of birth and eczema can be observed. Moreover, we examined the influence of sex of the infant and maternal history of allergic disease on the development of eczema and the diagnosis of AD in early infancy.

## Methods

### Study design and participants

We analyzed data obtained in the Japan Environment and Children’s Study (JECS), which is investigating the effects of environmental factors on children’s health. JECS is an ongoing nationwide government-funded prospective birth cohort study that was started in January 2011 [29, 30]. It is funded directly by the Ministry of the Environment, Japan, and involves collaboration among the JECS Programme Office (National Institute for Environmental Studies), the Medical Research Center for the JECS (National Center for Child Health and Development), and 15 regional centers [29, 30]. Pregnant women are contacted through cooperating health care providers and/or local government offices that issue the Maternal and Child Health Handbooks and those consenting to participate are registered. The population characteristics of the JECS cohort are highly representative of the Japanese population according to vital statistics published by the government [30].

### Ethics statement

JECS participants were recruited during their pregnancy by research coordinators after receiving face-to-face explanations of the overall aims of the study, and informed consent was obtained from all participants. JECS is conducted in accordance with the Declaration of Helsinki and applicable national regulations and guidelines. All procedures involving human subjects for the JECS protocol were reviewed and approved by the Ministry of the Environment’s Institutional Review Board on Epidemiological Studies (100,910,001) and the ethics committees of all participating institutions.

### Study data

**Fig. 1:**
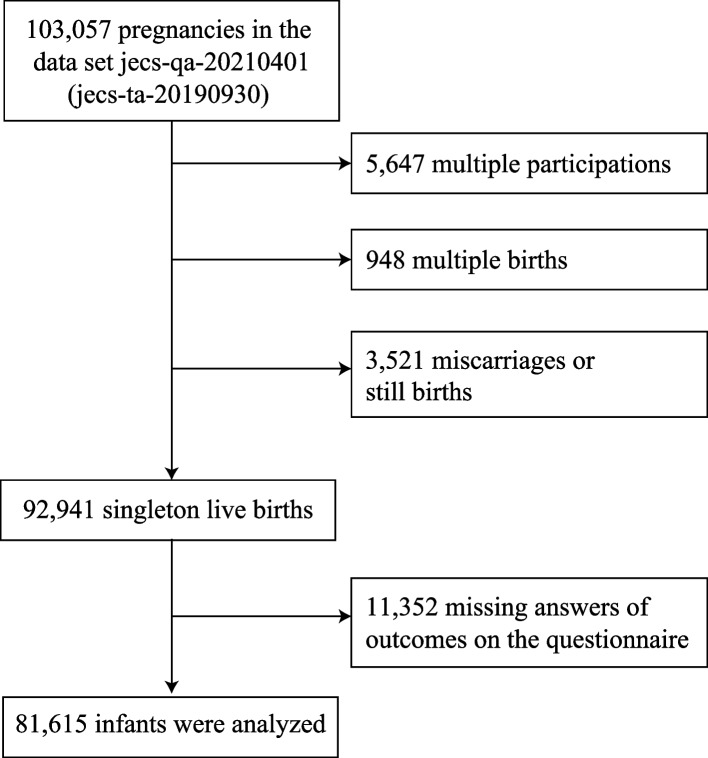
Participant flow diagram

This study used the jecs-qa-20210401 dataset (jecs-ta-20190930), which contains information on demographic factors, medical history, obstetric history, physical and mental health, lifestyle, occupation, housing situation, and socioeconomic status, obtained from participant responses to self-administered questionnaires or transcribed from the medical records by physicians, midwives, nurses, and/or research coordinators. The dataset contains the records of 103,057 pregnancies with information on mother–infant pairs. Of these, we excluded 5,647 multiple registrations (second or third registration of the same mother), 948 multiple births (twins or more), and 3,521 miscarriages or stillbirths. Among the remaining 92,941 singleton live births from unique mothers, an additional 11,352 were excluded due to unreturned questionnaires or missing response to items on the infant’s skin status or physician-diagnosed AD. Finally, we analyzed the data for 81,615 infants (Fig. 1).

### Exposure classification

First, we considered the month of birth as an exposure factor in our analysis. Then, we used season of birth for analysis. In a previous study, we found the highest incidence of AD in 3-year-olds born in October–December and the lowest incidence in those born in April–June [20]. Therefore, in this study, we classified seasons as spring (April–June), summer (July–September), autumn (October–December), and winter (January–March).

### Outcomes

Information on dermatitis was collected by distributing questionnaires to the caregivers (mainly mothers) of the infants at 1 month, 6 months, and 1 year after delivery. Although various definitions of AD and eczema have been reported [31], the present study refers to “eczema” as judged by the parents based on the symptoms they observed and to “AD” as diagnosed by a physician. We analyzed four outcomes reported on: eczema at 1 month of age, eczema at 6 months of age, eczema at 1 year of age, and physician-diagnosed AD reported at 1 year of age. Eczema at 1 month of age was defined as an affirmative response to the question, “Has your baby ever had a rash on the face, head, or neck or around the ear during the past month after birth?” Eczema at 6 months and eczema at 1 year of age were both defined as an affirmative response to the question, “Has your child ever had an itchy rash that was coming and going for at least 2 months?”, which was based on an item from the ISAAC questionnaire targeting 6–7-year-olds, the Japanese translation of which has been validated [23, 24]. Physician-diagnosed AD reported at 1 year of age was defined as an affirmative response to the question, “Has your child ever been diagnosed by a physician as having AD?”.

### Statistical analysis

To identify the association between season of birth and the four outcomes, we performed logistic regression analysis [32] and determined the 95% confidence intervals (CIs). Multiple testing correction was performed using the Benjamini–Hochberg method [33], and the false discovery rate was set at less than 0.05. Then, in multivariable logistic regression analysis, we adjusted for the following potential modifiers: maternal age, annual household income, maternal education level, maternal history of allergic disease (AD, bronchial asthma, allergic rhinitis, pollinosis, allergic conjunctivitis, and food allergy), maternal intake of vitamin D during pregnancy, maternal active smoking, maternal passive smoking, infant sex, gestational weeks, feeding method during the first month after birth, presence of older siblings, pet ownership, and registered regional center. These covariates were selected based on a previous study [22]. Vitamin D intake was obtained from a food frequency questionnaire [34] and was adjusted for energy intake using the residual method. Multicollinearity assessed using generalized variance-inflation factors greater than 10 was not detected.

To examine whether the relationship between season of birth and prevalence of eczema or AD differed by infant sex or maternal allergic history, parent-judged eczema and physician-diagnosed AD were analyzed separately after stratification of the cohort by sex and maternal history of allergic disease. SAS version 9.4 software (SAS Institute Inc., Cary, NC, USA) was used for all statistical analyses.

## Results

A total of 81,615 infants were analyzed in this study. Distribution by month of birth ranged from 7.0% to 10.2% (Table 1). Most infants were born in the summer, followed by the autumn, spring, and winter (29.0%, 25.1%, 23.5%, and 22.5%, respectively; Table 1). The overall prevalence of parent-judged eczema was 61.0% at 1 month, 33.0% at 6 months, and 18.7% at 1 year and that for physician-diagnosed AD was 4.3% up to 1 year of age.

**Table 1:** Demographic and obstetric characteristics of the infants

Characteristic	n (%) or mean (SD)
**Month of birth,** (*n* = 81,615)	
January	6,400 (7.8%)
February	5,736 (7.0%)
March	6,175 (7.6%)
April	6,308 (7.7%)
May	6,559 (8.0%)
June	6,292 (7.7%)
July	7,171 (8.8%)
August	8,190 (10.0%)
September	8,305 (10.2%)
October	7,691 (9.4%)
November	6,461 (7.9%)
December	6,327 (7.8%)
**Season of birth,** (*n* = 81,615)	
Spring (Apr–Jun)	19,159 (23.5%)
Summer (Jul–Sep)	23,666 (29.0%)
Autumn (Oct–Dec)	20,479 (25.1%)
Winter (Jan–Mar)	18,311 (22.4%)
**Maternal age (years),** (*n* = 81,611)	
< 25	7,105 (8.7%)
25–29	22,273 (27.3%)
30–34	29,405 (36.0%)
35–39	18,963 (23.2%)
≥ 40	3,865 (4.7%)
**Annual household income (JPY),** (*n* = 75,701)	
< 4 million	29,578 (39.1%)
4 – < 6 million	25,337 (33.5%)
≥ 6 million	20,786 (27.5%)
**Highest maternal educational level,** (*n* = 80,778)	
< 13	27,846 (34.5%)
13 – 15	34,581 (42.8%)
≥ 16	18,351 (22.7%)
**Maternal history of allergic disease,** (*n* = 81,203)	
Yes	40,799 (50.2%)
**Maternal intake of vitamin D during pregnancy (μg),** (*n* = 81,177)	
Mean (SD)	4.3 (2.8)
**Maternal active smoking during pregnancy,** (*n* = 80,587)	
Never-smokers	47,828 (59.4%)
Quit smoking before pregnancy	19,133 (23.7%)
Quit smoking during early pregnancy	10,563 (13.1)
Continued smoking	3,063 (3.8%)
**Maternal passive smoking during pregnancy,** (*n* = 80,979)	
Almost never	51,302 (63.4%)
Less than 1 day per week	9,712 (12.0%)
2–3 days per week	6,500 (8.0%)
4–6 days per week	3,877 (4.8%)
Every day	9,588 (11.8%)
**Infant sex,** (*n* = 81,613)	
Male	41,903 (51.4%)
Female	39,710 (48.7%)
**Gestational weeks (w),** (*n* = 81,459)	
Mean (SD)	38.9 (1.5)
**Feeding method during 1 month after birth,** (*n* = 81,350)	
Breastfeeding only	34,548 (42.5%)
Mixed feeding	45,740 (56.2%)
Infant formula only	1,062 (1.3%)
**Presence of older siblings at 1 month after birth,** (*n* = 81,615)	
Yes	42,695 (52.3%)
**Pet ownership,** (*n* = 80,830)	
Yes	18,290 (22.6%)
**Registered regional center,** (*n* = 81,615)	
Hokkaido	6,505 (8.0%)
Miyagi	6,700 (8.2%)
Fukushima	11,145 (13.7%)
Chiba	4,720 (5.8%)
Kanagawa	5,402 (6.6%)
Koshin	5,806 (7.1%)
Toyama	4,587 (5.6%)
Aichi	4,492 (5.5%)
Kyoto	3,247 (4.0%)
Osaka	6,268 (7.7%)
Hyogo	4,185 (5.1%)
Tottori	2,499 (3.1%)
Kochi	5,487 (6.7%)
Fukuoka	6,117 (7.5%)
South Kyushu/Okinawa	4,455 (5.5%)

*Abbreviations*: *JPY* Japanese yen, *SD* Standard deviation

**Fig. 2:**
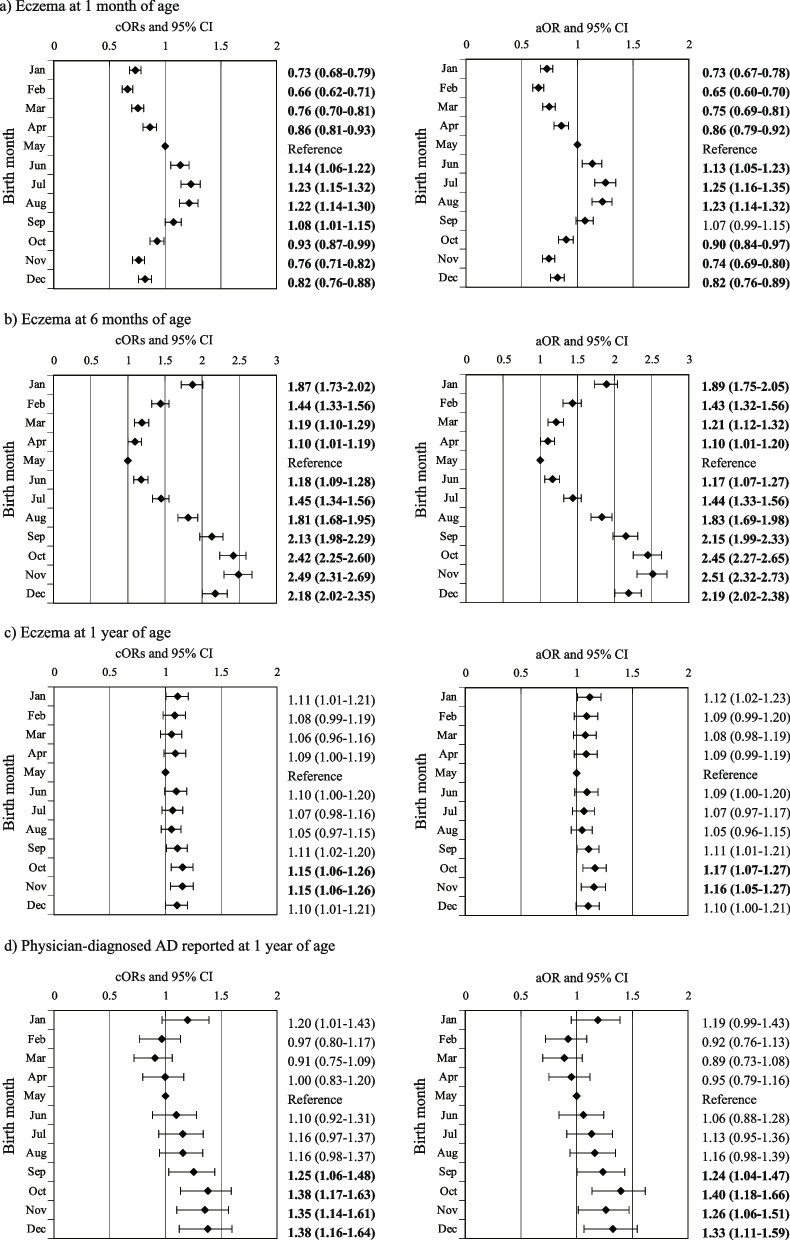
Odds ratios (95% CI) for outcomes in early infancy by birth month (*N* = 81,615). Adjusted for maternal age, annual household income, maternal educational level, maternal history of allergic disease, maternal intake of vitamin D during pregnancy, maternal active smoking during pregnancy, maternal passive smoking during pregnancy, infant sex, gestational weeks, feeding method, presence of older siblings, pet ownership, and registered regional center. Bold letters indicate statistically significant differences calculated using the Benjamini–Hochberg method.Abbreviations: AD, atopic dermatitis; aOR, adjusted odds ratio; cOR, crude odds ratio;95% CI, 95% confidence interval

Logistic regression analysis to examine the association between month of birth and each outcome revealed that, compared with infants born in May as a reference, infants born in July had the highest risk of eczema at 1 month (adjusted odds ratio [aOR], 1.25; 95% CI, 1.16–1.35; Fig. 2a), whereas infants born in November had the highest risk at eczema at 6 months (aOR, 2.51; 95% CI, 2.32–2.73; Fig. 2b) and those born in October had the highest risk at 1 year (aOR, 1.17; 95% CI, 1.07–1.27; Fig. 2c). Infants born in October also had the highest risk of physician-diagnosed AD (aOR, 1.40; 95% CI, 1.18–1.66; Fig. 2d).

When the infants were classified by season of birth, with those born in spring as a reference, infants born in summer had the highest risk of eczema at 1 month (aOR, 1.19; 95% CI, 1.14–1.24; Table 2a), whereas those born in autumn had the highest risks of eczema at 6 months (aOR, 2.19; 95% CI, 2.10–2.30; Table 2b) and at 1 year (aOR, 1.08; 95% CI, 1.02–1.14; Table 2c) and of physician-diagnosed AD up to 1 year of age (aOR, 1.33; 95% CI, 1.20–1.47; Table 2d).

**Table 2:** Odds ratios (95% CIs) for outcomes in early infancy by birth season (*N* = 81,615)

Season of birth	Case	Subtotal	Prevalence	cOR (95% CI)	aOR (95% CI)
**a) Eczema at 1 month of age**					
Spring (Apr–Jun)	12,017	19,159	62.7%	Reference	Reference
Summer (Jul–Sep)	15,726	23,666	66.4%	**1.18 (1.13–1.23)**	**1.19 (1.14–1.24)**
Autumn (Oct–Dec)	12,028	20,479	58.7%	**0.85 (0.81–0.88)**	**0.83 (0.80–0.87)**
Winter (Jan–Mar)	10,048	18,311	54.9%	**0.72 (0.69–0.75)**	**0.72 (0.69–0.75)**
**b) Eczema at 6 months of age**					
Spring (Apr–Jun)	4,668	19,159	24.4%	Reference	Reference
Summer (Jul–Sep)	8,218	23,666	34.7%	**1.65 (1.58–1.72)**	**1.66 (1.59–1.74)**
Autumn (Oct–Dec)	8,425	20,479	41.1%	**2.17 (2.08–2.27)**	**2.19 (2.10–2.30)**
Winter (Jan–Mar)	5,599	18,311	30.6%	**1.37 (1.31–1.43)**	**1.38 (1.32–1.45)**
**c) Eczema at 1 year of age**					
Spring (Apr–Jun)	3,514	19,159	18.3%	Reference	Reference
Summer (Jul–Sep)	4,390	23,666	18.5%	1.01 (0.97–1.07)	1.02 (0.96–1.07)
Autumn (Oct–Dec)	3,976	20,479	19.4%	**1.07 (1.02–1.13)**	**1.08 (1.02–1.14)**
Winter (Jan–Mar)	3,415	18,311	18.6%	1.02 (0.97–1.08)	1.04 (0.98–1.09)
**d) Physician diagnosed AD before 1 year of age**					
Spring (Apr–Jun)	731	19,159	3.8%	Reference	Reference
Summer (Jul–Sep)	1,036	23,666	4.4%	**1.15 (1.05–1.27)**	**1.18 (1.06–1.30)**
Autumn (Oct–Dec)	1,027	20,479	5.0%	**1.33 (1.21–1.47)**	**1.33 (1.20–1.47)**
Winter (Jan–Mar)	696	18,311	3.8%	1.00 (0.90–1.11)	1.00 (0.90–1.12)

Adjusted for maternal age, annual household income, maternal educational level, maternal history of allergic disease, maternal intake of vitamin D during pregnancy, maternal active smoking during pregnancy, maternal passive smoking during pregnancy, infant sex, gestational weeks, feeding method, food allergy symptoms at 1 month of age, presence of older siblings, pet ownership, and registered regional center. Bold letters indicate statistically significant differences calculated using the Benjamini–Hochberg method

*Abbreviations*: *AD* Atopic dermatitis, *aOR* Adjusted odds ratio, *cOR* Crude odds ratio, *95% CI* 95% Confidence interval

**Fig. 3:**
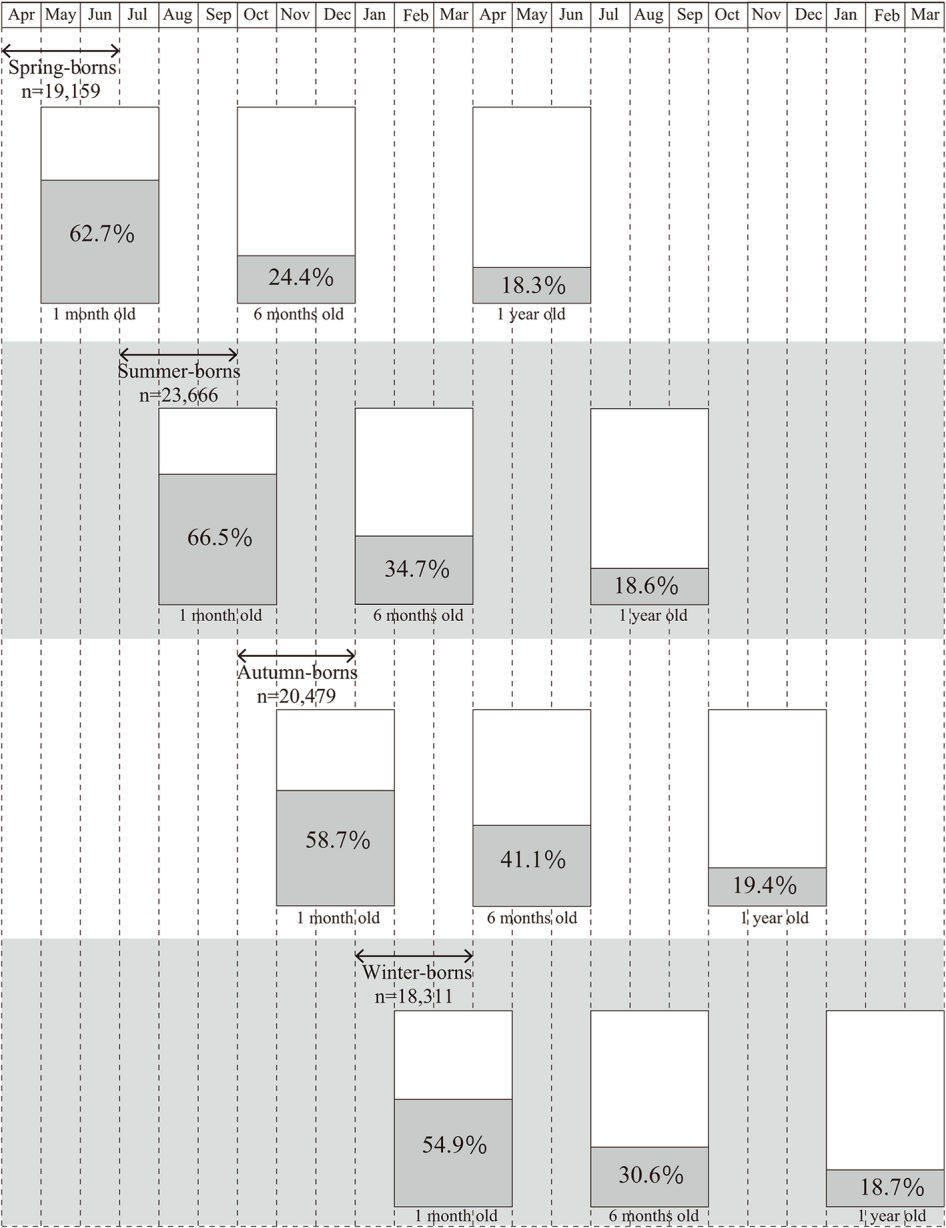
Prevalence of eczema by season of birth. This figure shows the season of information collection for eczema in infants of the same age in months. At 6 months of age, for infants born in spring, information was collected in the autumn (October–December), while for infants born in autumn, information was collected in the spring (April–June). Thus, the seasons of information collection at 6 months and 1 year of age were opposite

Figure 3 shows the relationship between season of birth and prevalence of eczema at 1 month, 6 months, and 1 year of age. At 6 months old, for infants born in spring, information was collected in autumn. For infants born in autumn, information was collected in spring. Although eczema at 6 months and at 1 year of age are opposite with regards to the season of observation, the highest frequency was identified for infants born in autumn at both ages. Between 1 and 6 months of age as well as between 6 months and 1 year of age, transitions in parent-judged eczema according to season of birth were categorized as no symptoms at either time point (symptom-free), symptoms improving from the first to second time point (improvement), symptoms occurring between the first and second time points (onset), and symptoms of the same severity at both time points (persistent). The results showed that infants with persistent eczema at either time point examined were more likely to be born in autumn (Supplementary Table 1).

Finally, we stratified the results by sex and maternal history of allergic disease. Prevalence of the four outcomes in both sexes with a maternal history of allergic disease was higher than in those without such history: eczema at 1 month of age, 63.5% vs 58.5%; eczema at 6 months of age, 36.2% vs 29.7%; eczema at 1 year of age, 22.0% vs 15.5%; and physician-diagnosed AD, 5.6% vs 3.0%. When the groups with and without maternal history allergic disease were stratified by sex, prevalence was higher in boys than in girls for all four outcomes (Table 3).

**Table 3:** Prevalence of outcomes stratified by maternal history of allergic disease and infant sex

	Without maternal history of allergic disease				With maternal history of allergic disease			
	Boys		Girls		Boys		Girls	
	*n* = 20,715		*n* = 19,689		*n* = 20,981		*n* = 19,816	
	n	%	n	%	n	%	n	%
Eczema at 1 month of age	12,667	61.1%	10,986	55.8%	13,890	66.2%	12,017	60.6%
Eczema at 6 months of age	6,964	33.6%	5,050	25.6%	8,438	40.2%	6,318	31.9%
Eczema at 1 year of age	3,663	17.7%	2,593	13.2%	5,113	24.4%	3,852	19.4%
Physician-diagnosed AD before 1 year of age	762	3.7%	436	2.2%	1,419	6.8%	857	4.3%

*Abbreviation*: *AD* Atopic dermatitis

To determine the association between season of birth and physician-diagnosed AD up to 1 year of age, subgroup analysis stratified by infant sex and maternal history of allergic disease revealed that, in girls, the aOR was not significant among those without maternal history of allergic disease, but for girls with such maternal history, those born in autumn showed a significant aOR compared with those born in spring (Table 4). Intriguingly, for boys with maternal history of allergic disease, those born in summer or autumn had a higher risk of AD (summer: aOR, 1.36; 95% CI, 1.15–1.60; autumn: aOR, 1.37; 95% CI, 1.16–1.62).

**Table 4:** Odds ratios for physician-diagnosed AD by birth season stratified by maternal allergic history and sex

Season of birth	Case	Subtotal	Prevalence	cOR (95% CI)	aOR (95% CI)
**Boys without maternal history of allergic disease (*n* = 20,715)**					
Spring (Apr–Jun)	169	4,925	3.4%	Reference	Reference
Summer (Jul–Sep)	205	6,105	3.4%	0.98 (0.80–1.20)	0.98 (0.79–1.22)
Autumn (Oct–Dec)	231	5,102	4.5%	**1.34 (1.09–1.63)**	1.25 (1.01–1.55)
Winter (Jan–Mar)	157	4,583	3.4%	1.00 (0.80–1.25)	0.95 (0.75–1.19)
**Girls without maternal history of allergic disease (*n* = 19,689)**					
Spring (Apr–Jun)	101	4,632	2.2%	Reference	Reference
Summer (Jul–Sep)	120	5,702	2.1%	0.96 (0.74–1.26)	0.96 (0.72–1.26)
Autumn (Oct–Dec)	145	4,970	2.9%	1.35 (1.04–1.74)	1.29 (0.99–1.69)
Winter (Jan–Mar)	70	4,385	1.6%	0.73 (0.54–0.99)	0.72 (0.52–0.98)
**Boys with maternal history of allergic disease (*n* = 20,981)**					
Spring (Apr–Jun)	279	4,933	5.7%	Reference	Reference
Summer (Jul–Sep)	444	6,055	7.3%	**1.32 (1.13–1.54)**	**1.36 (1.15–1.60)**
Autumn (Oct–Dec)	399	5,271	7.6%	**1.37 (1.17–1.60)**	**1.37 (1.16–1.62)**
Winter (Jan–Mar)	297	4,722	6.3%	1.12 (0.95–1.33)	1.16 (0.97–1.38)
**Girls with maternal history of allergic disease (*n* = 19,816)**					
Spring (Apr–Jun)	180	4,573	3.9%	Reference	Reference
Summer (Jul–Sep)	262	5,683	4.6%	1.18 (0.97–1.43)	1.20 (0.98–1.48)
Autumn (Oct–Dec)	251	5,033	5.0%	**1.28 (1.05–1.56)**	**1.34 (1.09–1.64)**
Winter (Jan–Mar)	164	4,527	3.6%	0.92 (0.74–1.14)	0.97 (0.78–1.22)

Adjusted for maternal age, annual household income, maternal educational level, maternal intake of vitamin D during pregnancy, maternal active smoking during pregnancy, maternal passive smoking during pregnancy, gestational weeks, feeding method, presence of older siblings, pet ownership, and registered regional center. Bold letters indicate statistically significant differences calculated using the Benjamini–Hochberg method

*Abbreviations*: cOR Crude odds ratio, aOR Adjusted odds ratio, *95% CI* 95% Confidence interval

## Discussion

In this large prospective birth cohort, environmental factors indicated by month of birth were found to influence the prevalence of eczema and AD diagnosis in infants younger than 1 year of age. In particular, the prevalence of eczema was found to be higher among those born in the autumn. Moreover, the importance of male sex and maternal history of allergic disease on the development of atopic eczema was evident. Because eczema is one of the earliest clinical manifestations of allergic disease and negatively affects quality of life [26, 28], our results may be significant from a preventive standpoint. Improved knowledge about the impact of season of birth on the development of eczema could inform future preventive measures to prevent skin barrier disruption, such as appropriate skin care from early infancy, and thereby reduce the risk of allergic diseases developing, especially in male infants with a maternal history of allergic disease.

It is well established that events during our early lives affect later life course trajectories [35, 36]. Previous studies have suggested that the initiation of antigen-specific responses can occur in utero, mediated by immunoglobulin E. [37]. It is difficult to examine in utero factors directly, however. Our results here do suggest the important involvement of early postnatal life in the development of eczema. Dry skin and the resulting skin barrier disruption are believed to be one of the non-allergic etiological factors of AD [38] because they lead to immunological dysregulation, which in turn may lead to allergens being sensitized epicutaneously, thereby inducing allergic inflammation of the skin. Our results suggest that the higher prevalence of eczema observed under certain climate conditions could be due to environmental triggering of subclinical disease in predisposed infants.

In Japan, children born in spring (the reference season in this study) spend their first few months in warm, humid weather, which results in moist skin, whereas those born in autumn spend their first few months in cold, dry weather, which can result in dry skin. They are also mainly at home in conditions that dry the skin. Winter in Japan, with its low indoor humidity and cold outside temperatures, is associated with a double negative influence on the skin barrier. Thus, changes in skin condition in the first few months of life could play an important role in triggering allergic skin inflammation and ultimately affect the prevalence of AD. Previous studies showed that the sex of the child was associated with the development of allergic diseases in childhood [6–8]. Moreover, mothers with AD may transmit it to their children [4, 5]. In the present study, the prevalence of physician-diagnosed AD was higher in boys with a maternal allergic history irrespective of season of birth (Table 3).

Infants born in the autumn in Japan tend to spend early infancy in a season with little sunshine. A previous study found higher levels of 25-hydroxy vitamin D and immunosuppressive cytokine IL-10 in the blood of infants born in spring than in those born in winter [39]. In line with this, low exposure to ultraviolet light and subsequently lower levels of vitamin D and/or IL-10 may explain the association between season of birth and development of AD. Vitamin D suppresses allergic sensitization by promoting Fop3^+^ regulatory T cells [40]. Indeed, reduced vitamin D and low fish intake by mothers during pregnancy have been correlated with increased incidence of AD in their children [41, 42]. Several trials have shown that vitamin D supplementation improves the clinical symptoms of AD [43–45]. In our analysis, we adjusted the data for the amount of dietary vitamin D intake during pregnancy. Therefore, we speculate that the influence of month of birth on the development of eczema in early infancy may be dependent on the vitamin D produced through exposure to sunshine in the children.

It is well recognized that the prevalence of AD is higher in infants born in autumn than in those born in spring [14, 15, 17–20]. A recent systematic review and meta-analysis also indicated that AD was significantly associated with autumn and winter births compared with spring births in the northern hemisphere [46]. However, our results show that eczema or rash was more prevalent at 1 month of age in infants born in the summer compared with those born in other seasons. Thus, the eczema seen at this age may be much more influenced by the season of observation. In Japan, hot and humid conditions in summer are associated with the prevalence of eczema in 1 month olds [47–49]. In our study, the prevalence was particularly high in 1 month olds (61.0%) compared with older infants, probably because eczema at this age shows a high degree of clinical heterogeneity, similar to other forms of eczema such as seborrheic dermatitis, intertrigo, and diaper dermatitis. In addition, about 60% of infants who had symptoms of eczema at 6 months no longer showed such symptoms at 1 year of age, which is a similar findings to that of previous Japanese studies where atopic dermatitis observed in infants younger than 6 months old remitted with age [50]. Although there are differences in prevalence between 6 months and 1 year of age, both time points show a higher prevalence among children born in autumn.

This study has several strengths. First, the prospective design of JECS, with the regular return of questionnaires, minimizes recall bias concerning the child’s AD. Also, JECS has a well-powered sample size, which enabled adjustment for various confounders in logistic regression analysis. The dataset covers about 45% of live births within the study areas, and the characteristics of the participants are similar to those of other women according to the Japanese vital statistics data, so the cohort is considered representative of the Japanese population [30]. We also studied the effect of possible confounders.

Nonetheless, this study has some limitations. Presence of eczema was determined from responses on a self-reported parental questionnaire. The rather broad question used might result in overestimates of eczema prevalence through the inclusion of other entities such as allergic contact dermatitis, and differences in the wording of survey questions at 1 month and at 6 months and 1 year of age might have an effect as well. We identified AD based on caregiver-reported physician diagnosis at 1 year of age, and the prevalence might differ if the outcome assessment were to be conducted using data collected from the medical records. Also, the reported diagnosis was not necessarily made by an allergy specialist. Furthermore, genetic polymorphisms known to be associated with the development of AD, such as filaggrin [51], could not be considered in this study.

A combination of multiple indicators, such as use of therapeutic agents and eczema characteristics, is considered to be the most confirmatory for the diagnosis of AD [31]. Because diagnosing AD in infants under 1 year of age is difficult, it is necessary to clarify those infants who are more susceptible to seasonal exposure, taking into account genetic polymorphisms and other factors. It is also necessary to clarify whether our results observed in Japan are generalizable to children in other parts of the world.

## Conclusions

Our results indicate that eczema and diagnosis of AD are more prevalent at age 6 months and 1 year in infants born in autumn than in those born in spring. However, at 1 month of age, eczema is more prevalent in infants born in summer. Thus, eczema in 1-month-olds may be influenced by the season of observation. The climate in early infancy seems to affect skin condition and ultimately influence the development of AD. Our results suggest that caregivers of male infants with a maternal history of allergic disease, and particularly those born in autumn, should pay careful attention to the infant’s skin.

## Supplementary Information

**Additional file 1:** **Supplemental Table 1.** Number (%) of childrenborn in each season stratified by transition in eczema status between time pointsin infancy.

## Data Availability

Data are unsuitable for public deposition due to ethical restrictions and the legal framework of Japan. It is prohibited by the Act on the Protection of Personal Information (Act No. 57 of 30 May 2003, amendment 9 September 2015) to publicly deposit data containing personal information. Ethical Guidelines for Medical and Health Research Involving Human Subjects enforced by the Japan Ministry of Education, Culture, Sports, Science, and Technology and the Ministry of Health, Labour and Welfare also restrict the open sharing of epidemiological data. All inquiries about access to data should be sent to: jecs-en@nies.go.jp. The person responsible for handling enquiries sent to this e-mail address is Dr. Shoji F. Nakayama, JECS Programme Office, National Institute for Environmental Studies.

## Abbreviations

AD: Atopic dermatitis
aOR: Adjusted odds ratio
cOR: Crude odds ratio
CI: Confidence interval
JECS: Japan Environment and Children’s Study
