# Supplementary material for: Season of birth and atopic dermatitis in early infancy: results from the Japan Environment and Children’s Study
Source: BMC Pediatr. 2023 Feb 15;23:78. doi: 10.1186/s12887-023-03878-6 (PMC9930333; doi:10.1186/s12887-023-03878-6)
Supplement: Supplementary file 1 — Additional file 1: Supplemental Table 1. Number (%) of childrenborn in each season stratified by transition in eczema status between time pointsin infancy. [file 12887_2023_3878_MOESM1_ESM.docx]

Supplementary Materials for

**Season of birth and atopic dermatitis in early infancy: Results from the Japan Environment and Children’s Study**

Akiko Tsuchida, Toshiko Itazawa, Kenta Matsumura, Hiroshi Yokomichi, Zentaro Yamagata, Yuichi Adachi, Hidekuni Inadera, and the Japan Environment and Children’s Study Group

Supplemental Table 1. Number (%) of children born in each season stratified by transition in eczema status between time points in infancy.

|  | From 1 to 6 months of age | From 6 months to 1 year of age |
| --- | --- | --- |
| **Spring-born (n=19,159)** |  |  |
| Symptom-free | 5869 (30.6%) | 12955 (67.6%) |
| Improvement | 8622 (45.0%) | 2690 (14.0%) |
| Onset | 1273 (6.6%) | 1536 (8.0%) |
| Persistent | 3395 (17.7%) | 1978 (10.3%) |
| **Summer-born (n=23,666)** |  |  |
| Symptom-free | 5,858 (24.8%) | 14,299 (60.4%) |
| Improvement | 9,590 (40.5%) | 4,977 (21.0%) |
| Onset | 2,082 (8.8%) | 1,149 (4.9%) |
| Persistent | 6,136 (25.9%) | 3,241 (13.7%) |
| **Autumn-born (n=20,479)** |  |  |
| Symptom-free | 5,762 (28.1%) | 11,390 (55.6%) |
| Improvement | 6,292 (30.7%) | 5,113 (25.0%) |
| Onset | 2,689 (13.1%) | 664 (3.2%) |
| Persistent | 5,736 (28.0%) | 3,312 (16.2%) |
| **Winter-born- (n=18,311)** |  |  |
| Symptom-free | 6,388 (34.9%) | 11,605 (63.4%) |
| Improvement | 6,324 (34.5%) | 3,291 (18.0%) |
| Onset | 1,875 (10.2%) | 1,107 (6.0%) |
| Persistent | 3,724 (20.3%) | 2,308 (12.6%) |
